# New ligation independent cloning vectors for expression of recombinant proteins with a self-cleaving CPD/6xHis-tag

**DOI:** 10.1186/s12896-016-0323-4

**Published:** 2017-01-05

**Authors:** Marco Biancucci, Jazel S. Dolores, Jennifer Wong, Sarah Grimshaw, Wayne F. Anderson, Karla J. F. Satchell, Keehwan Kwon

**Affiliations:** 1 Department of Microbiology-Immunology, Feinberg School of Medicine, Northwestern University, 303 E. Chicago Avenue, Ward 6-205, Chicago, IL 60611 USA; 2 Infectious Diseases Group, J. Craig Venter Institute, 9714 Medical Center Drive, Rockville, MD 20850 USA; 3 Center for Structural Genomics of Infectious Diseases, Feinberg School of Medicine, Northwestern University, Chicago, IL USA; 4 Department of Biochemistry and Molecular Genetics, Feinberg School of Medicine, Northwestern University, Chicago, IL USA; 5 Present address: Northwestern Memorial Hospital, Chicago, IL USA; 6 Present address: Indiana University, Bloomington, IN USA

**Keywords:** Self-cleavable tag, Protein purification, Cysteine Protein Domain, Ligation-independent cloning (LIC), InsP6

## Abstract

**Background:**

Recombinant protein purification is a crucial step for biochemistry and structural biology fields. Rapid robust purification methods utilize various peptide or protein tags fused to the target protein for affinity purification using corresponding matrices and to enhance solubility. However, affinity/solubility-tags often need to be removed in order to conduct functional and structural studies, adding complexities to purification protocols.

**Results:**

In this work, the *Vibrio cholerae* MARTX toxin Cysteine Protease Domain (CPD) was inserted in a ligation-independent cloning (LIC) vector to create a C-terminal 6xHis-tagged inducible autoprocessing enzyme tag, called “the CPD-tag”. The pCPD and alternative pCPD/*ccdB* cloning vectors allow for easy insertion of DNA and expression of the target protein fused to the CPD-tag, which is removed at the end of the purification step by addition of the inexpensive small molecule inositol hexakisphosphate to induce CPD autoprocessing. This process is demonstrated using a small bacterial membrane localization domain and for high yield purification of the eukaryotic small GTPase KRas. Subsequently, pCPD was tested with 40 proteins or sub-domains selected from a high throughput crystallization pipeline.

**Conclusion:**

pCPD vectors are easily used LIC compatible vectors for expression of recombinant proteins with a C-terminal CPD/6xHis-tag. Although intended only as a strategy for rapid tag removal, this pilot study revealed the CPD-tag may also increase expression and solubility of some recombinant proteins.

**Electronic supplementary material:**

The online version of this article (doi:10.1186/s12896-016-0323-4) contains supplementary material, which is available to authorized users.

## Background

Ligation-independent cloning (LIC) represents a robust and efficient cloning technique for recombinant proteins [1]. This cloning method exploits the 3’-5’ exonuclease activity of T4 DNA polymerase, which creates extended segments of complementary DNA of the amplified gene to facilitate the annealing with the target linearized vector. This procedure does not require classical endonuclease restriction enzyme digestion or in vitro ligation. LIC is suitable for high - throughput (HT) cloning platforms used to produce large numbers of proteins without considering restriction cleavage sites for each gene of interest. LIC is often used for rapid insertion of PCR products where HT cloning for recombinant protein production plays a fundamental role for the characterization of hundreds of proteins [2, 3].

Fusion tags play significant roles in enhancement of expression and solubility, and purification of target proteins. A hexahistidine tag (6xHis-tag) is one of the most frequently used fusion tags for affinity purification of recombinant proteins. Besides the 6xHis-tag, large soluble affinity tags, such as glutathione S-transferase (GST), maltose-binding protein (MBP), thioredoxin, the transcription termination anti-termination factor NusA or small ubiquitin-like modifier (SUMO) are often used to increase the solubility of recombinant proteins [4]. However, these affinity/solubility tags have to be removed after purification in order to conduct functional studies or crystallization [5]. Although large soluble tags enhance soluble expression of recombinant proteins, sometimes they cause misfolded and non-functional target proteins. The much smaller 6xHis-tag is a frequently used peptide tag for affinity purification, but it has been reported that the highly-charged tag can negatively affect protein crystallization and/or functional characterization [6, 7].

In order to remove any fusion tag, a protease cleavage site is frequently incorporated at the junction between the target protein and the affinity/solubility tag. Proteases commonly used to specifically cleave fusion tags include thrombin, SUMO protease, Factor X, and Tobacco Etch Mosaic Virus (TEV) protease. The efficiency of proteolytic processing can sometimes be complicated by steric hindrance or by unfavorable residues close to the cleavage site. Additionally, large-scale protein production can require a relatively large amount of the protease, which may limit the final protein yield and significantly increase the purification cost. Overall, affinity/solubility-tag removal adds another layer of complexity and expense to any purification process.

In the last decade, several self-cleaving tags have been developed. These special fusion tags possess an inducible activity, which can be triggered upon binding small molecules or conformational changes induced by particular buffer conditions. Shen A. et al. [8] demonstrated that the 6xHis-tagged Cysteine Protein Domain (CPD) of *Vibrio cholerae* could be fused at the C-terminus of the target protein and be used as self-cleavage fusion system. This technology was also developed independently by Northwestern University [9, 10]. CPD is a highly conserved domain of the large *V. cholerae* Multifunctional-Autoprocessing Repeats-in-Toxin (MARTX) toxin [11]. Once translocated by the toxin into the eukaryotic cytosol, the CPD is activated to autoproteolyze the toxin by inositol hexakisphosphate (InsP6), releasing several cytotoxic effector domains [11]. CPD cleaves after leucine residues located in accessible loops, which are able to occupy the hydrophobic pocket of S1 site [12, 13]. Although, only a leucine residue is strictly required for CPD autoprocessing, leucine residues located between small residues are preferred substrates [12, 13]. Since InsP6 is absent in bacteria cells, self-cleaving CPD fusion systems have been used as versatile tool for expression and purification of several proteins [12–14]. Moreover, the 6xHis-tagged CPD was demonstrated to improve expression, solubility, and stability of fusion proteins although the vectors used standard restriction enzyme cloning [8].

In this work, we report a new vector designated pCPD that incorporate sequences for a C-terminal 6xHis-tagged CPD (the CPD-tag) in-frame with a LIC site. We successfully tested two different self-cleaving CPD fusion proteins and compared the same proteins purified with a conventional TEV protease-cleavable 6xHis-tag. Additionally, pCPD was further modified by the insertion of a coupled cell division B (*ccdB*) cassette as a negative selection marker to create a vector more suitable for vector preparation and high throughput LIC. The pCPD/*ccdB* vector was used for HT cloning and demonstrated to improve success rates for both expression and solubility after removing the CPD-tag from recombinant proteins derived from a HT pipeline.

## Methods

### Enzymes and reagents

Restriction enzymes and Gibson Assembly^®^ cloning reagents were obtained from New England Biolabs and polymerases were obtained from Life Technologies. TOP10 competent cells and InsP6 (also known as phytic acid) were obtained from Sigma-Aldrich. Other common chemicals, antibiotics, and growth media components were from Sigma-Aldrich or Thermo Fisher.

Bacterial expression vectors for production of recombinant proteins pMCSG7, pMCSG53, and pMCSG58 were obtained from Dr. Andrzej Joachimiak of the Midwest Center for Structural Genomics (MCSG). The vector pMCSG7 consists of a LIC site and encodes for an N-terminal TEV cleavable 6xHis-tagged protein [15]. The vector pMCSG53 is an upgraded version of pMCSG7 that also carries the rare codon tRNA genes *argU* and *ileX* [16]. The vector pMCSG58 is similar to pMCSG53 except it encodes for a C-terminal 6xHis-tagged protein [16]. Proteins were expressed in kanamycin-resistant BL21(DE3)MAGIC *E. coli* strain. The pMagic plasmid also provides a second copy of the rare codon tRNA gene *argU* ([17] and A. Joachimiak, personal communication).

### New vectors for expression of CPD fusion proteins

#### Insertion of CPD into of pMCSG58 vector

DNA corresponding to coding sequence for amino acids L3428-G3637 of the *V. cholerae rtxA* gene (*vc1451*, geneID 2613957) was amplified from purified *V. cholerae* N16961 chromosomal DNA using Pfx50 DNA polymerase, forward primer 5’-CTTTAAGAAGGAGTCTCTCCCGGGGCAGCATTAGCGGATGG-3’ and reverse primer 5’-GATGATGATGGTGGTGAGCCCCACCTTGCGCG-3’. The vector pMCSG58 was digested with SmaI restriction enzyme and purified. 3 pmol of PCR product and 1 pmol of linearized vector were incubated with Gibson Assembly^®^ master mix for 1 h at 50°C [18]. 1 μl of the Gibson cloning reaction was transformed into TOP10 electro-competent cells. The final plasmid pCPD was confirmed to be accurate by DNA sequencing.

#### Incorporation of *ccdB*/*Cm*^R^ cassette

pCPD was modified by incorporating *ccdB* cassette at the cloning site to prepare pCPD/*ccdB* for a HT LIC platform. The cassette of *ccdB* and *Cm* ^*R*^ (chloramphenicol resistant gene *cat*) in pDONR221 was amplified by polymerase chain reaction using primers, 5’-TTAACTTTAAGAAGGAGTCTCTCCCGGG CTTCATCTGGATTTTCAGC’ and 5’-TTTTTCCATCCGCTAATGCTGCCCCGGG TTATCGAGATTTTCAGGAGCTAA-3’. The 5’-end sequences of the oligonucleotides are pCPD vector specific, the 3’-end sequences are *ccdB*/*Cm* ^*R*^ cassette specific. The SmaI cleavage sequence (CCCGGG) was incorporated into the center of the oligonucleotides. The vector pCPD was digested with SmaI. Both amplified *ccdB*/*Cm* ^*R*^ cassette and digested vector were gel-purified. 20 μl Gibson assembly^®^ reaction with 30 fmol purified insert and vector backbone was performed as previously reported [18]. 20 μl of assembly reaction was transformed into *ccdB* survival TM T1 Phage-resistant *E. coli*. Transformants were plated on LB-agar containing 100 μg/mL ampicillin and 25 μg/mL chloramphenicol. Selected transformants were grown in 2xYT with antibiotics for plasmid preparation. The final plasmid pCPD/*ccdB* was confirmed by DNA sequencing.

### Cloning, expression, and purification of MLD_Vv_

DNA sequences corresponding to the membrane localization domain (MLD, NP_759056.1, amino acids Q3591-L3674) was amplified from purified *V. vulnificus* CMCP6 chromosomal DNA using forward primer 5’-GTCTCTCCCATGCAAATCTTTACGGTGCAAGAGCT-3’ and reverse primer 5’-TGCTGCCCCCAACGCACTGGTGACCTGCT-3’ and the products were cloned into SmaI-digested pCPD by LIC [1]. The plasmid was confirmed to be accurate by DNA sequencing and then transformed into *E. coli* BL21(DE3)/MAGIC. Culture was grown in Terrific Broth supplemented with 100 μg/mL ampicillin and 30 μg/mL of kanamycin at 37 °C until OD_600_ = 0.8 and then induced with 1 mM isopropyl-β-D-thiogalactoside (IPTG) at 25 °C for ≈ 18 h. Bacteria were harvested by centrifugation, re-suspended in buffer A1 (10 mM Tris pH 8.3, 500 mM NaCl, 0.1% Triton X-100, 1 mg/ml lysozyme, 5 mM β-mercaptoethanol (BMe) and lysed by sonication. After centrifugation at 30,000x*g* for 30 min, the soluble lysate was incubated with Ni-NTA resin (GE Healthcare) for 30 min at 4 °C. The Ni-NTA resin was recovered onto a gravity column and washed with buffer B1 (10 mM Tris pH 7.5, 500 mM NaCl, 50 mM imidazole) followed by elution in the same buffer with 500 mM imidazole (buffer C1). Protein was exchanged into buffer A1 (without Triton X-100) using a PD10 desalting column (GE Healthcare). The protein solution was incubated with 100 μM InsP6 for 1 h at 25 °C under gentle agitation and then loaded again onto the gravity column containing Ni-NTA (GE Healthcare), the flow through fraction containing the MLD was collected. Purified proteins were analyzed by SDS-PAGE.

### KRas expression and functional characterization

#### *Cloning*, *expression*, *and purification KRas*

KRas full-length (fl) and KRas catalytic domain (residues 1–169) were amplified by PCR from a plasmid template containing the full-length KRas gene [19]. In order to insert KRas-fl and KRas_1–169_ into pMCSG7 and pCPD, three different set of primers were designed and used: KRas-fl-pCPD-FWD 5’-CTTTAAGAAGGAGTCTCTCCCATGACTGAATATAAACTTGTGGTAGTTGGAGCTGG-3’ - KRas-fl-pCPD-REV 5’-CCGCTAATGCTGCCCCCATAATTACACACTTTGTCTTTGACTTCTTTTTCTTC-3’; KRas_1–169_-pMCSG7-FWD 5’-CCTGTACTTCCAATCCAATGCTATGACTGAATATAAACTTGTGGTAGTTGG-3’ - KRas_1–169_-pMCSG7-REV 5’-CCGTTATCCACTTCCAATTTACTACTTTTCTTTATGTTTTCGAATTTCTCGAACTAATG-3’; KRas_1–169_-pCPD-FWD 5’-CTTTAAGAAGGAGTCTCTCCCATGACTGAATATAAACTT GTGGTAGTTGG-3’ - KRas_1–169_-pCPD-REV 5’-CCGCTAATGCTGCCCCCTTTTCTTTATGTTTTCGAATTTCTCGAACTAATG-3’) for Gibson Assembly^®^ cloning method. The vectors pMCSG7 and pCPD were digested with SspI or SmaI, respectively. PCR products (KRas_1–169_-pMCSG7, KRas-fl-pCPD and KRas_1–169_-pCPD) and linearized vectors were gel-purified. 10 μl of Gibson Assembly^®^ master mix, 1 pmol pMCSG7 or pCPD linearized vectors and 3 pmol the respective PCR products were incubated for 1 h at 50 °C. 1 μl of the Gibson Assembly^®^ reaction mix was transformed into TOP10 electro-competent cells. Plasmids were confirmed to be accurate by DNA sequencing and then transformed into *E. coli* BL21(DE3)/MAGIC. All KRas fl proteins were expressed as described above for MLD. KRas_1–169_-CPD and KRas_1–169_ were purified as described here. Bacteria were harvested by centrifugation, re-suspended in buffer A1 (50 mM Tris pH 8.3, 500 mM NaCl, 10 mM MgCl_2_, 0.1% Triton X-100, 5 mM BMe) and lysed by sonication. After centrifugation at 30,000x*g* for 30 min, the soluble lysates were loaded onto a 5-ml HisTrap column using the ÄKTA Purifier system (GE Healthcare). The column was washed with buffer B1 (10 mM Tris pH 7.5, 500 mM NaCl, 10 mM MgCl_2_, 50 mM imidazole) followed by elution in the same buffer with 500 mM imidazole (buffer C1). Proteins were further purified by size-exclusion chromatography (SEC) (Superdex 200 (26/60), GE Healthcare) in buffer D1 (10 mM Tris–HCl pH 7.5, 500 mM NaCl, 10 mM MgCl_2_, 5 mM BMe). The 6xHis-tag of KRas_1–169_ expressed from pMCSG7 was cleaved overnight with TEV protease and further purified by removal of uncleaved protein using a HisTrap column (GE Healthcare). The KRas_1–169_-CPD fusion protein was incubated with 100 μM InsP6 (Sigma Aldrich) for 1 h at 25 °C under gentle agitation. The protein sample treated with InsP6 was loaded onto a HisTrap column (GE Healthcare) and the flow through fraction containing KRas_1–169-GAAL_ was collected. Purified proteins were analyzed by SDS-PAGE. The Coomassie stained gel was scanned and band intensity of each recombinant protein was quantified using the NIH ImageJ v1.49 software.

#### *Cloning*, *expression*, *and purification of GAP*-*334*

Sequence of GAP-334 (residues 712–1046) was amplified from a plasmid containing the p120 sequence found in the *KRAS* Entry and *RAS* Pathway Clone Collection (Leidos Biomedical Research, Inc.) using the following primers: FWD 5’-CCTGTACTTCCAATCCAATGCTATGGAAAAAATCATGCCAGAAGAAGAG-3’ and REV 5’-CCGTTATCCACTTCCAATTTACCTGACATCATTGGTTTTTGTATAC-3’. The PCR product was cloned into pMCSG7 as described for KRas_1–169_ above. Plasmids were confirmed to be accurate by DNA sequencing and then transformed into *E. coli* BL21(DE3)/MAGIC. GAP-334 protein was expressed and purified as described above for KRas_1–169_, except all buffers were adjusted to pH 8.3. Purified protein was analyzed by SDS-PAGE.

#### *KRas Single*-*turnover GTP hydrolysis*

Single-turnover GTP hydrolysis analysis was performed as previously described [20]. All buffers were made phosphate-free by dialysis with 2 U of nucleoside phosphorylase and 0.5 mM of inosine. Briefly, for intrinsic hydrolysis, 20 μM of Ras was used. For GAP-mediated hydrolysis GAP-334 was used at final concentration of 0.2 μM and 0.1 μM, in order to have a GAP:Ras ratio of 1:100 and 1:200 respectively. FlippiU purified from vector pRSET FLIPPi-5u (Addgene) as previously described [21] was used to detect in real-time the amount of inorganic phosphate released in solution by the GTP hydrolysis [21]. The ratio of fluorescence emission 480/530 nm was measured with an excitation of 435 nm on SpectraMax M3 (Molecular Devices). Data were converted to phosphate concentration using a standard curve. Data were fit in GraphPad Prism 6.0 to a one-phase exponential association curve to determine the *k*_obs_. All reactions were performed in triplicate.

### Compare HT expression and protein solubility

#### PCR amplification of target ORFs and cloning into two vectors

Forty protein targets representing the next forty proteins or subdomains in the HT structural determination pipeline of the CSGID were selected. Two LIC expression vectors, pCPD/*ccdB* and pMCSG53/*ccdB* were used for protein expression and target open reading frames (ORFs) were amplified by PCR. In order to clone into pCPD/*ccdB* vector, PCR primers were designed such that gene specific sequences were followed by vector specific regions 5’-GTCTCTCCC, and 5’-TGCTGCCCC in forward primer and reverse primer, respectively. For pMCSG53/*ccdB*, vector specific region sequences, 5’-TACTTCCAATCCAATGCG and 5’-TTATCCACTTCCAATG were added to forward and reverse primers. PCR amplification of ORFs was performed using a standard PCR condition.

The PCR products for pCPD were treated with T4 DNA polymerase in the presence of dTTP. The LIC vector, pCPD/*ccdB* was digested with SmaI (5U/μL DNA) to remove *ccdB* and treated with T4 DNA polymerase in the presence of dATP, After T4 DNA polymerase treatment, 0.02 pmol of vector DNA and 0.04 pmol of insert DNA were mixed and annealed at 22 °C for 30 min as previously described [15]. 2 μL of the reactant was transformed into 25 μL of chemically competent *E. coli* DH5α using the heat shock method.

Cloning into pMCSG53/*ccdB* used a modified method of cloning for pMCSG7 as previously described [15]. PCR product and vector backbone from linearized pMCSG53/*ccdB* were treated with T4 DNA polymerase in the presence of dCTP and dGTP, respectively. Annealing of vector backbone and PCR and transformation into DH5α were performed as described above for cloning targets into pCPD.

#### Protein Expression Screening and Cleavage

The forty plasmids for each vector system were transformed to *E. coli* BL21(DE3)/MAGIC. Cells were grown in 1 mL 2xYT in a 96-well block at 37 °C at 900 rpm using a multitron shaker. Once cells reached an OD_600_ = 0.8, gene expression was induced by adding IPTG to a final concentration of 1 mM and incubating at 25 °C for 18–20 h. Cells were pelleted by centrifugation at 2,200x*g* for 30 min at 4 °C and stored at −80 °C. Cell pellets were thawed and resuspended in lysis buffer (50 mM Tris–HCl, 5 mM imidazole, 300 mM NaCl, 1mM dithiothreitol (DTT) pH 7.8 at 4 °C) and lysed by adding PopCulture Reagent (EMD-Novagen). Aliquots of cell lysates were transferred to a new 96-well plate and the remaining were centrifuged at 2,200x*g* for 1 h to separate the soluble fraction. The soluble fractions were transferred to a 96-well plate. Whole lysate and supernatant fractions were processed on the LabChip GXII (Perkin Elmer, Waltham, MA) to examine expression and solubility level of N-terminal 6xHis-tag fused target proteins. C-terminal CPD-tag fused recombinant proteins were purified using Ni-NTA agarose resin in a 96-well block as previously described [22]. The recombinant proteins were eluted in 200 μL elution buffer (50 mM Tris–HCl, 200 mM imidazole, 300 mM NaCl, 1mM DTT pH 7.8 at 4 °C). The purified proteins were exchanged into a lysis buffer and concentrated using a 96-well Multiscreen filter plate with an Ultracel-10 membrane (EMD-Millipore, Billerica, MA). The CPD-tag of the recombinant proteins were activated by adding InsP6 to a final concentration of 100 μM and incubating for 2 h at room temperature. The cleaved 6xHis-tagged CPD was removed by adding 25 μL Ni-NTA agarose resin and incubated for 30 min at 4 °C. The target proteins were transferred into a new 96-well plate. Whole lysates and supernatants and purified and cleaved proteins were examined HT capillary electrophoresis (LabChip GXII) and/or NuPAGE with Coomassie blue staining (Additional file 1: Figure S1). As previously reported [23], the level of expression and solubility of recombinant proteins were scored as four categories: 0 (undetectable), 1 (low), 2 (medium), and 3 (high) (Additional file 2). The scale is approximately, 0: <2%, 1: 2-10%, 2: 5–10%, 3: >10%. The purity percentage was obtained directly from LabChip HT software.

## Results and discussion

### Design and development of self-cleavage LIC/CPD fusion system

**Fig. 1:**
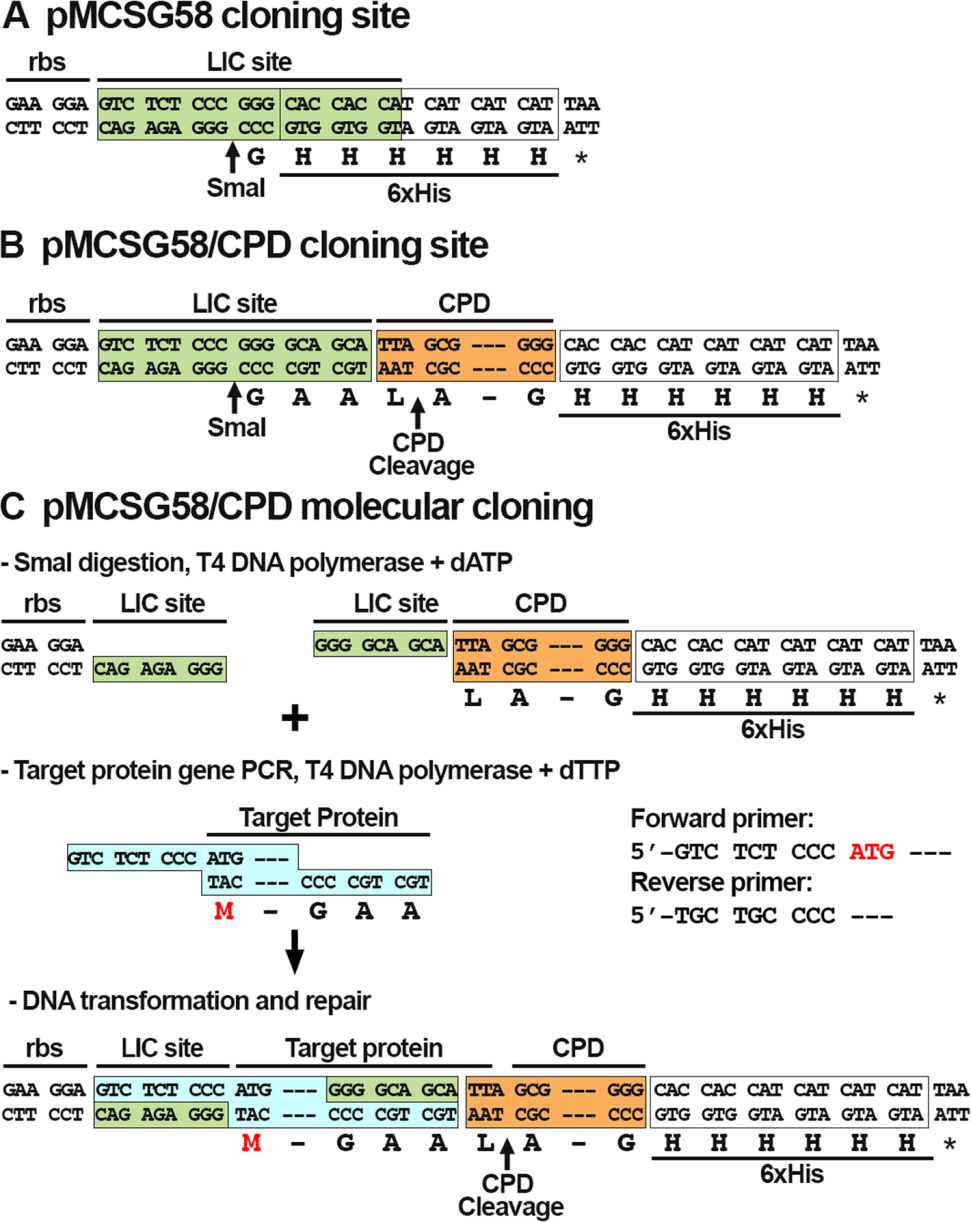
Design and cloning strategy of pCPD. **a** Sequence of LIC site in pMCSG58; **b** Sequence of LIC site in pCPD; **c** Molecular cloning strategy for the pCPD vector. After SmaI digestion linearized pCPD is treated with T4 polymerase and dATP. Target protein is amplified by PCR using LIC primers. PCR product is treated with T4 polymerase and dTTP. Both vector and PCR product are mixed for annealing and then transformed into competent cells

The *cpd* DNA sequence was amplified with LIC primers specific for SmaI-digested the vector pMCSG58 [16], which was used as backbone for the creation of the LIC/CPD fusion system (Fig. 1a). pMCSG58 is a bacterial expression vector that encodes a C-terminal 6xHis-tag and additionally contains a LIC site and *argU* and *ileX* genes for rare codon tRNAs [16]. The forward primer was designed with extra nucleotides in order to restore the LIC site and SmaI restriction site after the insertion of *cpd* sequences. In the newly created vector pCPD, the sequence for the LIC site is in frame with codons for the CPD followed by a 6xHis-tag (Fig. 1b). This vector pCPD is thus designed to express recombinant proteins with a CPD-tag at the C-terminus both to mimic its natural activity and because it is currently unknown whether N-terminal CPD-tag can efficiently autoprocess its own C-terminus [12]. DNA sequences of a target protein can be cloned into pCPD by LIC with T4 DNA polymerase or by Gibson Assembly^®^ [18] (Fig. 1c) or other commercial direct cloning methods. The protein fusion resulting from expression of DNA sequences cloned into pCPD will have four additional residues Gly-Ala-Ala-Leu at the junction between the target protein and the CPD (Fig. 1c). These four residues represent the encoded sequence for LIC and provide a Leu and a flexible linker for insertion into the CPD active site. The addition of InsP6 to a purified fusion protein will trigger activation of CPD proteolytic activity and the recombinant protein will be cleaved after the Leu (Fig. 1c).

**Fig. 2:**
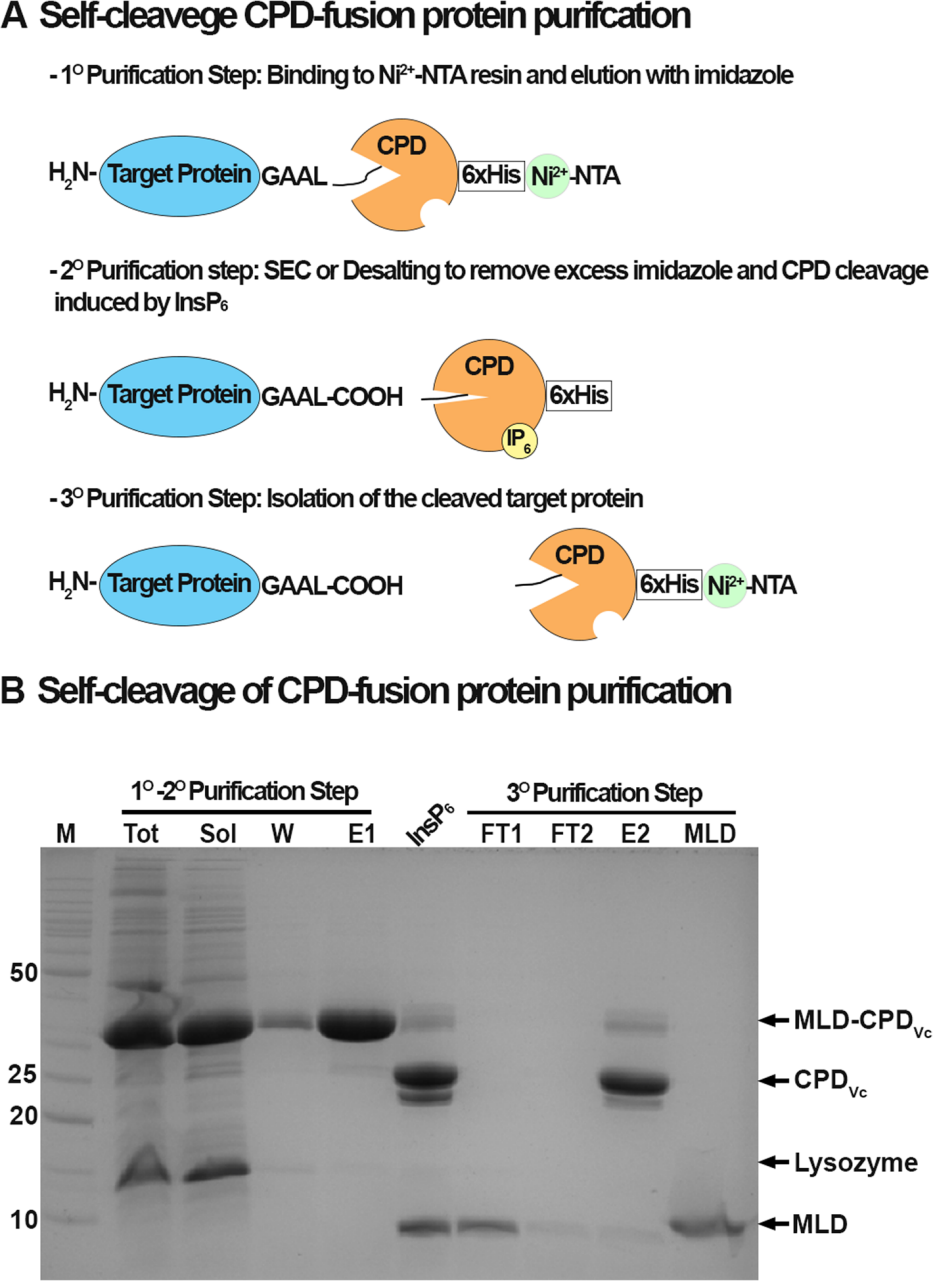
Purification strategy of autoprocessing CPD fusion-tag. **a** Self-cleaving CPD-fusion protein purification; **b** Purification of MLD. Marker (M), Total proteins (Tot), Soluble proteins (Sol), Washing step with 50 mM imidazole (W), Elution step with 500 mM imidazole containing MLD-CPD (E1), 1 h incubation with InsP6 (InsP6), First flow-through fraction containing MLD (FT1), Second flow-through fraction (FT2), Elution step with 500 mM imidazole containing CPD (E2), and previously purified MLD (MLD)

The C-terminal CPD-tag was previously proposed as a rapid single-step purification strategy for the 6xHis-tag removal while still bound on the initial column [8]. However, many biochemical studies require recombinant proteins with high purity and integrity. Thus, we sought to develop a purification protocol using a two-step removal/purification process rather than single step. For high efficiency of cleavage, the uncleaved CPD fusion protein is first eluted from Ni-NTA column followed by a second desalting or size exclusion step to remove imidazole and protein aggregates from the CPD protein-fusion (Fig. 2a). The CPD fusion-protein is then treated in solution with InsP6, triggering CPD autoprocessing and thereby releasing the target protein. The cleaved CPD-tag is separated from the target protein by an additional passage over a Ni-NTA column (Fig. 2a).

In order to test the feasibility of the system to purify small proteins, purification of the 83 amino acid MLD from the *V. vulnificus* MARTX toxin Ras/Rap1 Specific Protease (RRSP) [19, 24] was conducted. MLD is a 10 kDa protein while CPD is 27 kDa and as shown in the Fig. 2b, the fusion protein ran at the expected molecular weight on an SDS-polyacrylamide gel. Imidazole in the elution buffer was removed using a desalting column and the protein solution was adjusted to 100 μM InsP6. After an hour, the fusion protein was completely cleaved. The MLD was separated from the CPD-tag by incubating the protein solution with Ni-NTA resin and collecting the unbound fraction (Fig. 2b). As shown in the Fig. 2b, the MLD cleaved by CPD (MLD_GAAL_) ran at the expected molecular weight.

### Using pCPD vector for expression of highly soluble KRas

To test for successful purification of a eukaryotic protein, Ras, a human oncoprotein involved in the regulation of cell proliferation, differentiation and survival [25, 26]. Although recombinant Ras proteins are well expressed in *E. coli*, the final purification yield of the isoform KRas is low due to the formation of inclusion bodies [27]. To test whether our self-cleavage system could improve KRas yield, we cloned the full-length *KRAS* gene sequence or the sequence corresponding to the catalytic domain (KRas_1–169_) into pCPD. For comparison to an alternative expression system, the genes were also cloned into pMCSG7, which was previously used for KRas production [19]. The vector pMCSG7 has the LIC site and encodes for an N-terminal 6xHis-tag and TEV cleavage site [15].

**Fig. 3:**
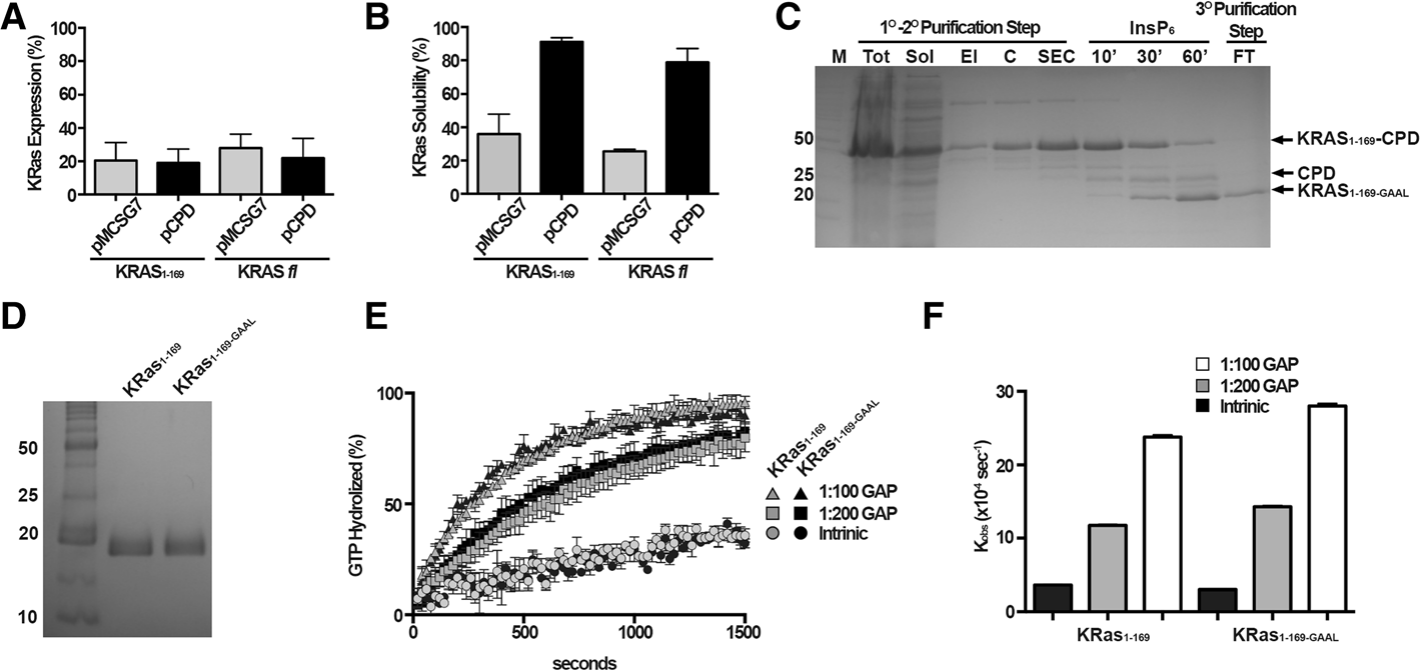
Expression and purification of KRas with pCPD and pMCSG7. **a**-**b** Expression and solubility of KRas_1–169_ and KRas fl. The amount of expressed and soluble proteins were analyzed by SDS-PAGE and measured with ImageJ software. Bars are the average of three independent biological replicates; **c** Marker (M), Total proteins (Tot), Soluble proteins (Sol), Elution step with 500 mM imidazole containing KRas_1–169_ -CPD (El), protein concentration step (C), SEC step (SEC), incubation time with InsP6, 10, 30 and 60 min respectively (10’-30’-60’), Flow-through fraction containing KRas_1–169 -GAAL_ (FT); **d** SDS-PAGE of KRas_1–169_ and KRas_1–169 –GAAL_; **e**-**f** Intrinsic and GAP mediated single-turnover GTP hydrolysis for KRas_1–169_ and KRas_1–169 –GAAL_. GAP-334 was used at two different molar ratios of 1:100 and 1:200 (GAP:KRas). Each curve and bar represent the average of three independent biological replicates

Although the protein expression for each construct was similar, KRas-CPD and KRas_1–169_-CPD were highly soluble (Fig. 3a and b). In particular, KRas_1–169_-CPD was three times more soluble than N-terminal 6xHis-tagged KRas_1–169_ (6xHis-TEV-KRas) expressed from the vector pMCSG7 and KRas-CPD was four times more soluble than N-terminal 6xHis-tagged KRas (Fig. 3b). KRas_1–169_-CPD and 6xHis-TEV-KRas_1–169_ were each purified to quantify the final purification yield and to check for function in a GTPase activity assay. Lysates containing each protein were loaded onto a Ni-NTA column and eluted in imidazole buffer as the first step of purification. Subsequently, KRas_1–169_-CPD and 6xHis-TEV-KRas_1–169_ were subjected to SEC. Elution fractions containing KRas_1–169_-CPD were pooled and treated with 100 μM InsP6. After 10 min, 30 min and 1 h, protein samples were collected to check CPD autoprocessing. After 1 h, the protein fusion was cleaved and the solution containing KRas_1–169-GAAL_ and the CPD-tag were passaged across a Ni-NTA column (Fig. 3c). KRas_1–169-GAAL_ was recovered into the flow-through fraction with a final yield of 35 mg/L. Separately, purified 6xHis-TEV-KRas_1–169_ was incubated overnight with TEV protease, then the protein solution was loaded onto Ni-NTA column and the flow-through fraction was collected, the final protein yield was 11 mg/L. Both KRas_1–169_ and KRas_1–169-GAAL_ were separated by SDS-PAGE showing high purity and expected similar molecular weight (Fig. 3d).

In order to compare the catalytic activities of the two different KRas proteins, GTPase activity was performed using FlippiU as a protein sensor for inorganic phosphate released in solution (Fig. 3f). KRas_1–169_ and KRas_1–169-GAAL_ showed the same magnitude of intrinsic and GAP-mediated GTP hydrolysis activities, suggesting that there were no functional differences between the proteins.

### Developing pCPD with a *ccdB* cassette for HT cloning

LIC vectors are usually prepared by restriction digestion and agarose-gel excision. Although the gel purification decreases background transformation after LIC, the digested vector is difficult to separate from the relaxed undigested vector. Therefore, even after agarose-gel purification, there is potentially still contamination with undigested vector, which can decrease the number of plasmids containing the target gene inserted, reducing the success rate in the HT cloning platform.

**Fig. 4:**
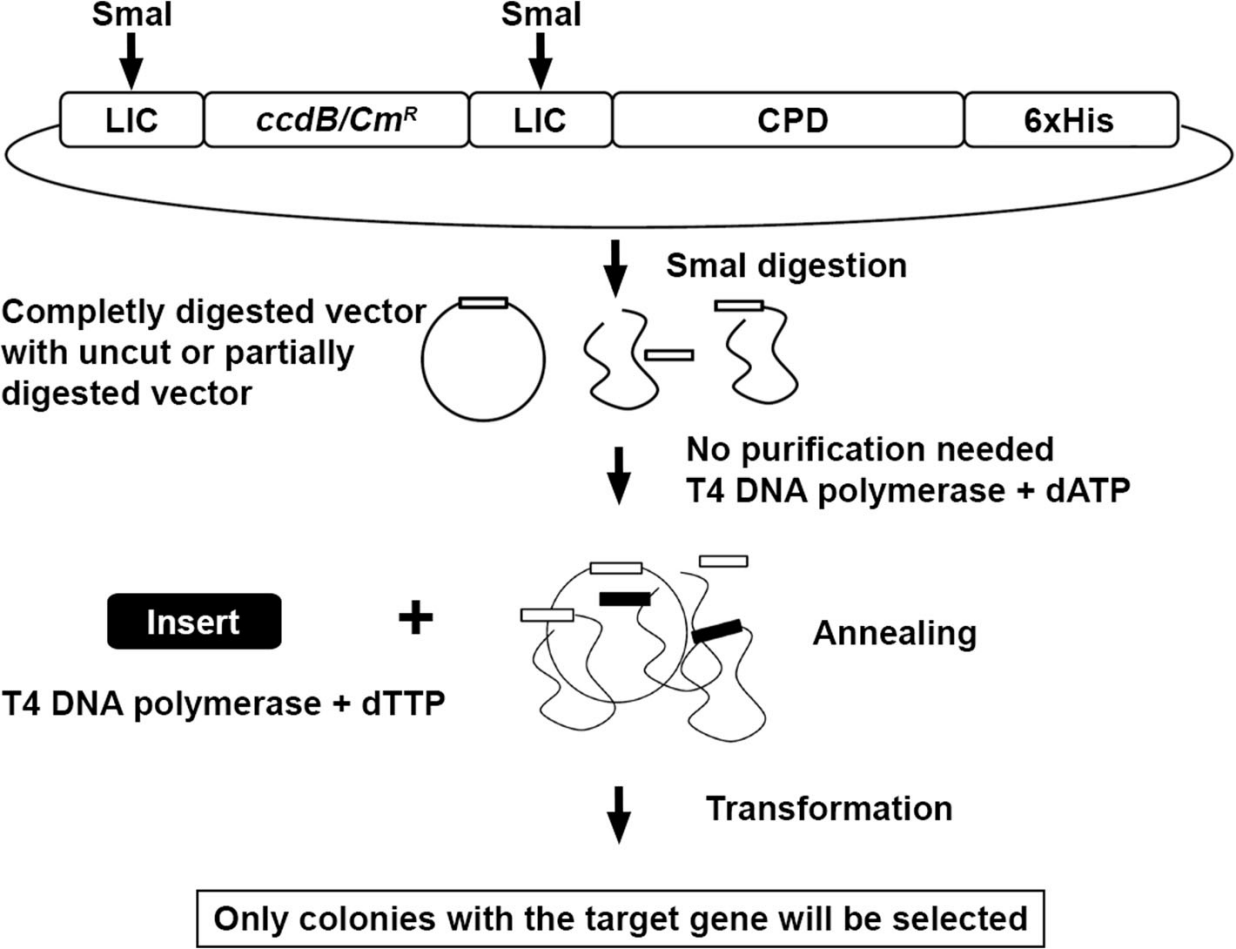
Molecular cloning strategy of pCPD/*ccdB*

In order to improve cloning efficiency for the purpose of HT cloning, the vector pCPD was further modified by insertion of the *ccd*B gene, which encodes for a cytotoxin that targets DNA gyrase [28] (Fig. 4). The *ccdB* gene then acts as a negative selection marker, because transformed cells with incomplete or undigested vector will not survive after LIC due to the presence of *ccdB* [29–31]. Thus, the advantage of the *ccdB* insertion is that only fully digested plasmids that have successfully incorporated the target gene insert are transformable. To insert *ccdB*, pCPD was digested with SmaI, and the PCR amplified 1823 bp *ccdB* and chloramphenicol resistance gene cassette (*ccdB*/*Cm* ^*R*^) were ligated into the site. In order to avoid loss of the LIC site, two SmaI sites were incorporated into the PCR primers, such that SmaI digestion of the final pCPD/*ccdB* vector is identical to that from unmodified pCPD when linearized (Fig. 4).

### Use of pCPD increases expression and solubility fusion proteins

To more broadly investigate the applicability of this method, we utilized resources of the National Institute of Allergy and Infectious Diseases (NIAID) Center for Structural Genomics of Infectious Disease (CSGID, http://csgid.org), which conducts HT cloning, protein production, and protein crystallization as an NIAID community resource. The next 40 CSGID target genes from this pipeline were selected to perform an unbiased pilot analysis of the impact the new pCPD vector could have on HT strategies. These 40 proteins include 36 unique bacterial proteins and 4 subdomains from 13 different species, although the majority (22 proteins) are proteins related to the type 2 secretion system (T2SS) from *Klebsiella pneumoniae*. The target genes were amplified by PCR, inserted into pCPD/*ccdB*, and expressed from the T7 promoter. As a control, the same fragments were cloned and expressed using pMCSG53/*ccdB*, a vector that expresses proteins fused to an TEV cleavable N-terminal 6x-His tag similar to pMCSG7 except that the plasmid also carries the rare tRNA codon genes *ileX* and *argU* (similar to pCPD/*ccdB* derived from pMCSG58) [16].

**Fig. 5:**
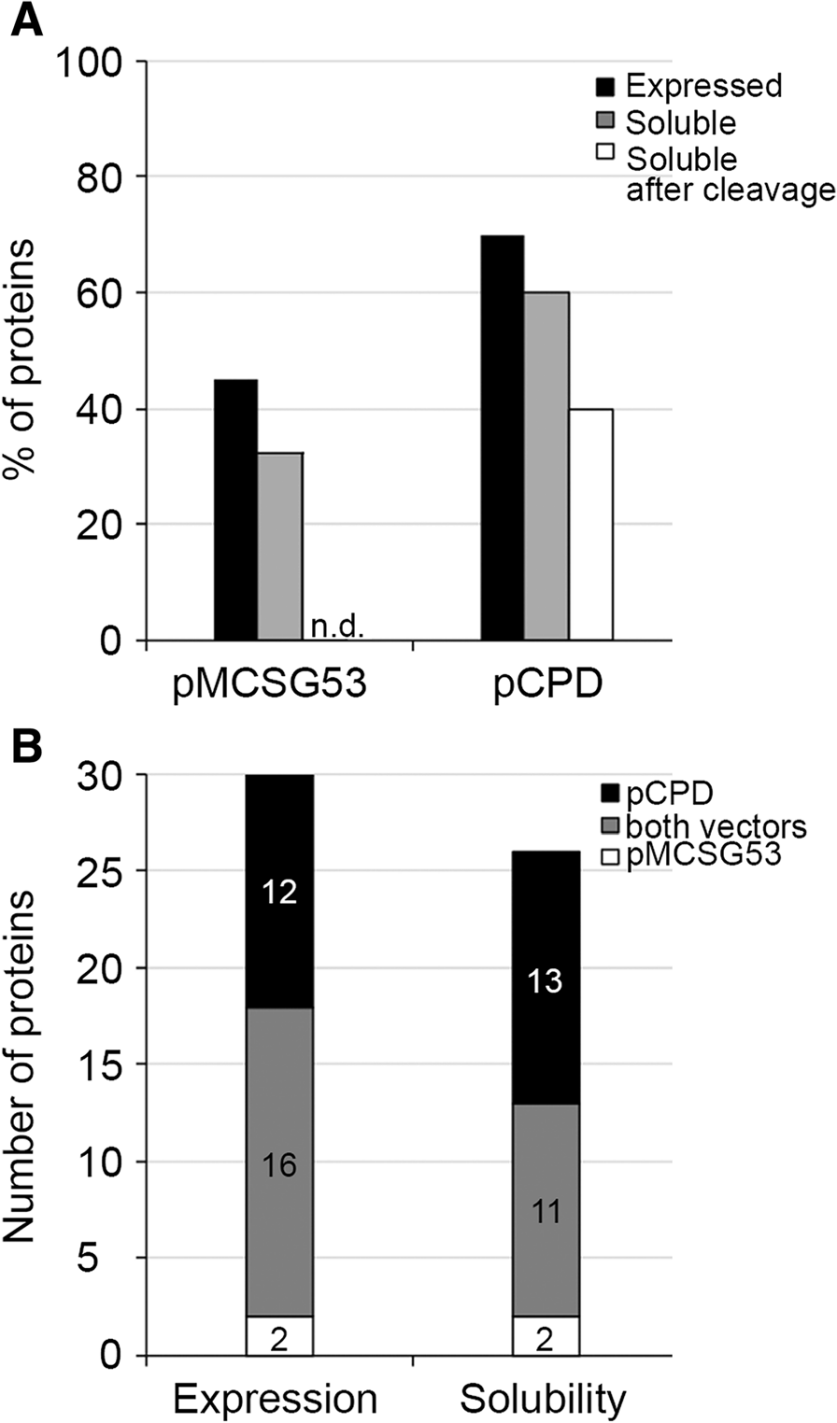
Expression and solubility of 40 bacterial proteins produced with pCPD and pMCSG53. **a** Percentage or **b** Number of expressed and soluble proteins resulting from expression of 40 selected target proteins from the indicated vector or vectors. n.d., not done

For all the recombinant proteins produced, the whole cell lysate and the supernatant fluid from induced cells were analyzed by SDS-PAGE and scored (Additional files 1 and 2). Twenty-eight (70%) of the CPD-fusion proteins were expressed when the genes were induced in *E. coli* compared to only 18 (45%) of those expressed with a TEV cleavable N-terminal 6xHis-tag (Fig. 5a). Sixteen HT targets were successfully expressed from both vectors, 12 were expressed exclusively from pCPD, while two were expressed exclusively from pMCSG53 (Fig. 5b). In terms of solubility, out of the 28 expressed CPD-fusions proteins, 24 (60%) were soluble, while 13 of the 18 expressed N-terminal 6xHis-tagged proteins were found in the supernatant fluid after lysis (Fig. 5b). In addition, three of the 16 proteins that were expressed from both plasmids were soluble only when expressed fused with CPD (Fig. 5b). Thus, the CPD functions not only as an easy strategy to remove a 6xHis-tag, but also enhances the success rate of protein expression and solubility.

A major concern of the use of solubility tags is that proteins become insoluble upon tag removal. 16 of the 24 soluble CPD-fusions proteins were still soluble upon processing (Table 1). Thus, an HT strategy employing only pCPD would be expect to return 40% soluble proteins with tags removed. This is comparable or slightly better than an HT strategy dependent only on pMCSG53, which returned 33% (13/40) soluble proteins with the 6xHis-tag. Since removal of small 6xHis-tags does not generally result in protein insolubility, cleavage by TEV protease was not done and would not be expected to change this frequency. Of note, some proteins that were soluble from each vector were not the same (Additional file 2), such that an HT strategy that would employ both expression vectors in a side-by-side approach would be predicted to yield 53% soluble protein. Notably, since conducting this pilot, two structures deposited to PDB (ID 5DO8 [32] and 5F7S [33]) have been determined for proteins purified from pCPD, indicating that the presence of the 4 aa on the C-terminus does not interfere with crystallization. For these two proteins, CPD autoprocessing was performed at 4 °C overnight. This, together with previously tested conditions including 0.4 M urea [12], suggest that the removal of the CPD-tag can be achieved within a broad range of temperature from 4 °C to 37 °C and for different amount of time from 1 min to overnight without compromising the CPD activity.

**Table 1:** Summary of success frequencies in expression of 40 recombinant proteins in *E. coli* using two expression vectors, pCPD and pMCSG53^a^

	pCPD		pMCSG53		Combined	
Total target proteins	40		40		40	
Expressed proteins in *E. coli*	28	(70%)	18	(45%)	30	(75%)
Soluble proteins	24	(60%)	13	(33%)	26	(65%)
Purified recombinant proteins	20	(50%)	n.d.		n.d.	
Soluble cleaved proteins	16	(40%)	n.d.		21^b^	(53%)

^a^n.d., not done

^b^Combined soluble proteins expressed using pMCSG53 (uncleaved) and soluble cleaved proteins expressed using pCPD

## Conclusions

In this work, a new vector, pCPD, was developed for overexpression of recombinant proteins with a C-terminal fusion to CPD-tag inducible by InsP6 to remove itself by autoprocessing. This new vector has several advantages over previous CPD-fusion vectors that depended on classical restriction enzyme gene insertion [8]. First, the backbone vector pMCGS58 provided a site for easy insertion of genes for protein expression by LIC or by recombination methods such as Gibson Assembly® and rare codons genes *ileX* and *argU* for improved success in protein expression. The pCPD vector was initially tested for two target proteins that had previously proven difficult to purify due to small size and insolubility, MLD and KRas, respectively. Interestingly, pCPD was shown to increase significantly the solubility of KRas_1–169_. Intrinsic and GAP stimulated GTP hydrolysis of KRas_1–169-GAAL_ was equally active to KRas_1–169_ purified with an N-terminal 6xHis-tag. In addition, the developed purification protocol can be easily applied to a high yield-high purity, two-step production scale purification strategy of Ni-NTA followed by SEC. Strategies for single step purification and cleavage could also be developed and useful when protein yield and purity are not as essential as they are in structural determination and biochemical assessment.

The vector pCPD was then further modified to contain *ccdB* cassette in order to increase cloning efficiency. Using a pilot set of 40 proteins, the CPD-tag was unexpectedly shown to increase the rate of success for expression and solubility of recombinant proteins by 23% when compared with proteins expressed with a TEV protease cleavable 6xHis-tag. Thus, pCPD and pCPD/*ccdB* are excellent options both to be implemented as a first line approach to recombinant protein expression and solubility, or as a side-by-side alternative to standard TEV protease cleavable tags for successful recombinant protein production.

## Additional files

Additional file 1: Figure S1. Sixteen representative samples of proteins expressed from vector pCPD show diversity of expression, solubility and recovery after InsP6 induced autoprocessing. Numbers at top indicate CSGID IDP designation as listed in Additional file 2. Lanes for each sample represent Whole cell lysate (L), Soluble fraction from lysate (S), Purified fusion protein (P), and purified protein after CPD autoprocessing (P’). Figure was assembled from multiple SDS-polyacrylamide gels stained with Coomassie Blue. The scores at each stage and the total amount of purified protein after autoprocessing are shown below the gel figure. The amounts of total purified proteins from 1 mL cultures were determined from absorbance at 260 nm using a NanoDrop. (TIFF 830 kb)
Additional file 2: Table S1. List of proteins examined for expression and solubility. IDP# Identification protein number relative to CSGID targets (http://csgid.org/); Locus ID for NCBI; Expression: expressed proteins are highlighted in yellow (1-Low expression, 2-Medium expression, 3-High expression). Non-expressed proteins are highlighted in white; Solubility: soluble proteins are highlighted in yellow (1-Low solubility, 2-Medium solubility, 3-High solubility). Non-soluble proteins are highlighted in white (0-undetectable solubility); Purified: proteins purified by single step on Ni-NTA resin and desalting. Purified proteins are highlighted in yellow (1-Low solubility, 2-Medium, 3-High). Non-purified proteins are highlighted in white (0-undetectable); InsP6 cleavage: proteins cleaved by InsP6 CPD autoprocessing activation and purified by “negative” Ni-NTA purification step. Soluble proteins after the cleavage are highlighted in yellow (1-Low solubility, 2-Medium, 3-High). Proteins lost after InsP6-cleavage/Ni-NTA purification are highlighted in white (0-undetectable). (XLSX 16 kb)

## Abbreviations

6xHis: Hexahistidine tag
*ccd*B: Coupled cell division B
CPD: Cysteine Protein Domain
HT: High-throughput
InsP6: Inositol hexaphosphate
LIC: Ligation-independent cloning
MARTX: Multifunctional-Autoprocessing Repeats-in-Toxin
MLD: Membrane localization domain
Ni-NTA: Nickel-charged affinity resin
SEC: Size exclusion chromatography
TEV: Tobacco Etch Virus
