# Supplementary figures and images for: New ligation independent cloning vectors for expression of recombinant proteins with a self-cleaving CPD/6xHis-tag
Source: BMC Biotechnol. 2017 Jan 5;17:1. doi: 10.1186/s12896-016-0323-4 (PMC5216533; doi:10.1186/s12896-016-0323-4)

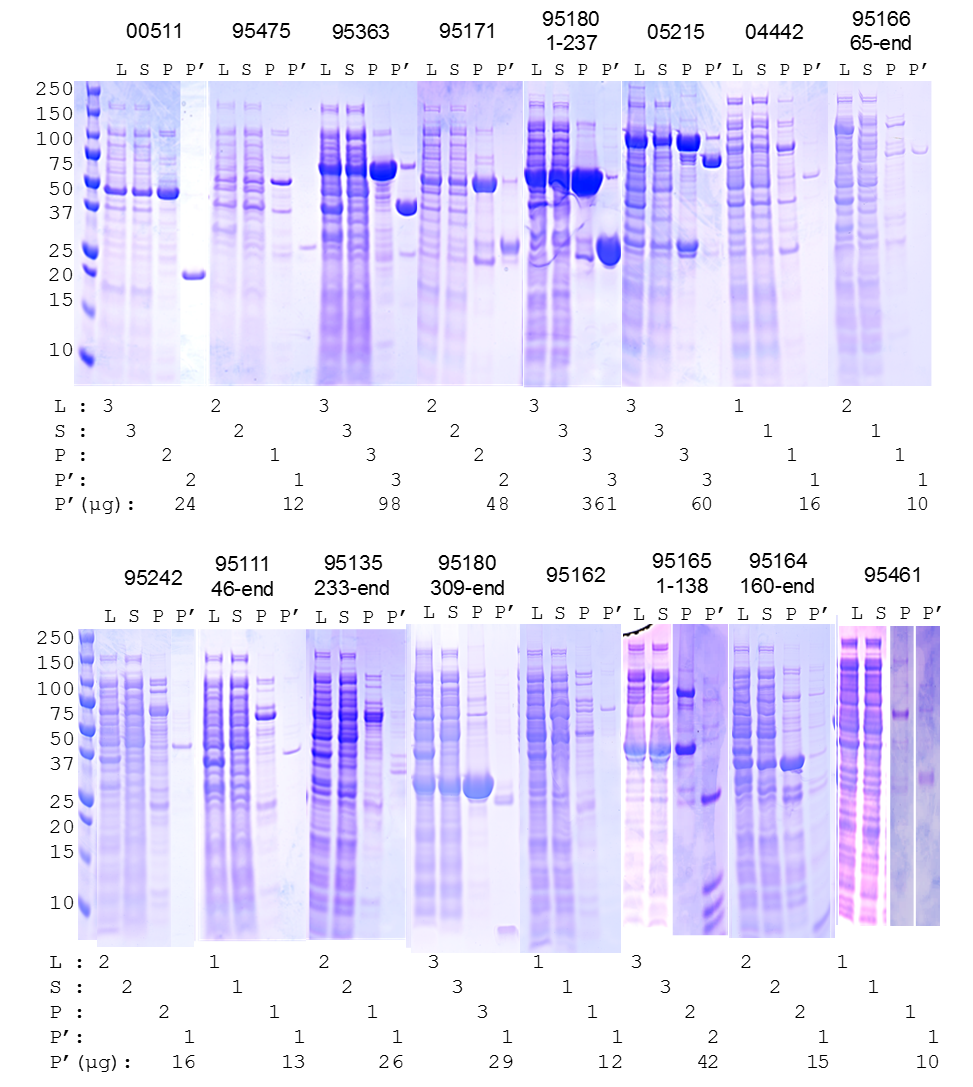

Supplement: Additional file 1: Figure S1. — Sixteen representative samples of proteins expressed from vector pCPD show diversity of expression, solubility and recovery after InsP6 induced autoprocessing. Numbers at top indicate CSGID IDP designation as listed in Additional file 2. Lanes for each sample represent Whole cell lysate (L), Soluble fraction from lysate (S), Purified fusion protein (P), and purified protein after CPD autoprocessing (P’). Figure was assembled from multiple SDS-polyacrylamide gels stained with Coomassie Blue. The scores at each stage and the total amount of purified protein after autoprocessing are shown below the gel figure. The amounts of total purified proteins from 1 mL cultures were determined from absorbance at 260 nm using a NanoDrop. (TIFF 830 kb) [file 12896_2016_323_MOESM1_ESM.tiff]
